# An Alarming Decline in the Nutritional Quality of Foods: The Biggest Challenge for Future Generations’ Health

**DOI:** 10.3390/foods13060877

**Published:** 2024-03-14

**Authors:** Raju Lal Bhardwaj, Aabha Parashar, Hanuman Prasad Parewa, Latika Vyas

**Affiliations:** 1 College of Agriculture, Sumerpur-Pali, Agriculture University, Jodhpur 342304, Rajasthan, India; 2 Agricultural Research Substation, Sumerpur-Pali, Agriculture University, Jodhpur 342304, Rajasthan, India; 3 Directorate of Extension Education, Maharana Pratap University of Agriculture and Technology, Udaipur 313001, Rajasthan, India

**Keywords:** biodiversity, micronutrient, nutrient density, traditional food, synthetic fertilizers

## Abstract

In the last sixty years, there has been an alarming decline in food quality and a decrease in a wide variety of nutritionally essential minerals and nutraceutical compounds in imperative fruits, vegetables, and food crops. The potential causes behind the decline in the nutritional quality of foods have been identified worldwide as chaotic mineral nutrient application, the preference for less nutritious cultivars/crops, the use of high-yielding varieties, and agronomic issues associated with a shift from natural farming to chemical farming. Likewise, the rise in atmospheric or synthetically elevated carbon dioxide could contribute to the extensive reductions in the nutritional quality of fruits, vegetables, and food crops. Since ancient times, nutrient-intense crops such as millets, conventional fruits, and vegetables have been broadly grown and are the most important staple food, but the area dedicated to these crops has been declining steadily over the past few decades and hastily after the green revolution era due to their poorer economic competitiveness with major commodities such as high-yielding varieties of potato, tomato, maize, wheat, and rice. The majority of the population in underdeveloped and developing countries have lower immune systems, are severely malnourished, and have multiple nutrient deficiency disorders due to poor dietary intake and less nutritious foods because of ignorance about the importance of our traditional nutrient-rich diets and ecofriendly organic farming methods. This critical review emphasizes the importance of balance and adequate nutrition as well as the need to improve soil biodiversity and fertility: those are main causes behind the decline in nutritional density. There is also emphasis on a possible way out of alleviating the decline nutritional density of food crops for the health and well-being of future generations.

## 1. Introduction

Globally, more than two billion people are suffering from micronutrient insufficiency, especially iodine, iron, folate, vitamin A, and zinc [1,2,3]. It is the main cause of premature deaths, morbidity, and retardation in the physical and mental growth of children [4]; in 2017, 11 million deaths and 255 million daily-adjusted life years (DALYs) could be attributed to malnutrition [5]. Since the 1940s, crop yield and the per-capita availability of foods have been continuously increasing due to intensive farming techniques, artificial fertilization, pesticides, irrigation, growing high-yielding varieties, and other environmental means, whereas malnutrition tends to increase incessantly due to disrupting the fine balance of soil life and decreasing the nutritional density and quality of the food crops. At present, people are overfed but undernourished due to consuming nutrient-poor diets [6,7]. It is quite difficult to obtain an equal concentration of nutrition from the food that was enjoyed before the pre-green revolution era. Important commercial high-yielding fruits such as apples, oranges, mango, guava, banana, and vegetables such as tomato and potato have lost their nutritional density by up to 25–50% or more during the last 50 to 70 years due to environmental, genetic, and field soil dilution factors [8]. Mayer et al. [9] reported that the elements except for phosphorus declined in the previous eighty years (1940 to 2019): sodium (52%), iron (50%), copper (49%), and magnesium (10%). The major reason for the variation appears to be that novel strains/varieties of crops have been introduced over the decades that produce additional yield, growth rate, and pest and disease resistance but go for lower levels of nutrients. Thus, declining nutrient concentrations in food products are most detrimental for future generations’ health. As a consequence, exploring the best management strategies that can solve the problems of the decline in the nutritional quality of foods is a major focus of the study, so that the sustainable health of the habitat can be achieved. Improving the consumption of nutrient-dense food crops is an important and possible way to tackle the global nutrient deficiencies and optimize the nutritional quality of these foods. There is a well-recognized need to achieve a better nutritional quality in foods with studies concurring that the pre-eminent way to achieve this is through the dual-purpose approach of making improvements to food systems as well as addressing health and nutrition goals [10]. For the verification of the study, we also considered various studies conducted in various parts of the world that focused on dietary diversification, supplementations of micronutrients [11,12], improving the nutritional quality of food, biofortification, soil fertility management, plant-breeding approaches [13], natural farming, reviving traditional foods [14], the production of nutrient-intense underutilized fruits and vegetables [15], beneficial soil microbial inoculation, and soil biodiversity [16,17]. The aim of this study was (i) to investigate the systematic decline in the nutritional quality of food crops, (ii) to assess the causes of declining nutrient density, and (iii) to identify the best management strategies for the maintenance of nutritional density in foods or to explore the available scientific evidence for enhancing the nutrient density and quality of fruits, vegetables, and food crops for the nutritional well-being of future generations.

A literature search was conducted for all articles indexed by Google, Web of Science, and Scopus up to 2022. The search strategies were completed using keywords including “food”, “food quality”, “nutritional dilution, food and nutrition”, “nutrient density”, “orgenoleptic quality of foods”, and “micronutrients depletion rate in soil and foods”. The keywords were amended slightly for each database. Sugar crops, food supplements, and all alcoholic beverages were excluded from the data acquisition. Through the established search strategy, 365 studies in total were initially assessed. Out of these, 200 closely related studies were considered for concluding the task, and a field survey was also conducted from 2017 to 2021 in which 1500 tribal farmers were interviewed to explore the changes in food habits, nutritional patterns, and the dietary and organoleptic qualities of different foods.

## 2. Nutrients’ Depletion Tendency of Foods

The nutrient exhaustion started long ago, but after 1900, the rate of dilution increased incessantly and after the green revolution exponentially. On the basis of available nutrition data, it was observed that in the past 70–80 years, the nutritional dilution rate was up to only 20%, whereas 80% dilution happened during the last 30–40 years. According to numerous studies [18,19,20,21] in many countries, the nutrient density and taste quality of fruits, vegetables, and foods crops have fallen extremely in the previous 50–70 years regarding sodium (29 to 49%), potassium (16 to 19%), magnesium (16 to 24%), calcium (16 to 46%), iron (24 to 27%), copper (20 to 76%), and zinc (27 to 59%). Mayer [22] observed declines in the nutrient levels of twenty vegetables from 1936 to 1991, including calcium (19%), magnesium (35%), and copper (81%), as well as in the nutrient levels of twenty fruits, including sodium (43%), magnesium(11%), iron(32%), copper (36%), and potassium (20%). Numerous scientists [23] found a significant percentage decline in the mineral content of thirteen fruits and vegetables during 1963 to 1992 in the U.S., including calcium (29%), magnesium (21%), potassium (6%), phosphorus (11%), and iron (32%), as well as a decrease in the mineral levels of twenty fruits and vegetables during in the last fifty-one years (1936 to 1987) in Britain: calcium (19%), magnesium (35%), sodium (43%), potassium (14%), phosphorus (6%), iron (22%), and copper (81%). Thomas [18] reported theatrical losses in copper (76%) and zinc (59%) during 1940 to 1991 and 1978 to 1991 in different vegetables, respectively. Similarly, Alae-Carew et al. [24] reported 50% less availability of water and use of saline water (3–4dS m−1) significantly reduces yield and nutritional quality of fruits. Likewise, pronouncements in [7,22] showed a consistent decline in the quantity of protein (6%), calcium (16%), phosphorus (9%), iron (15%), vitamin A (18%), riboflavin (38%), and vitamin C (15%) in 43 different fruits and vegetables over the past half century. Jack [25] reported a fall in nutrients such as calcium (26.5%), iron (36.1%), vitamin A (21.4%) and vitamin C (29.9%) in vegetables from 1975 to 1997. Different vegetables lost a greater part of their iron, including cauliflower (60%), collard greens (81%), mustard greens (51.3%), onions (56%), and watercress (88.2%), and vitamin A in broccoli (38.3%), cauliflower (68.3%), collard greens (41.2%), and parsley (38.8%). A sharp decline rate was observed in calcium, with 57.4 percent in lemons, 58.8 percent in pineapples, and 65 percent in tangerines during 1975 to 2001. The authors also observed from available nutritional data that the phosphorus levels dropped in different fruits such as apples (30%), bananas (52.4%), oranges (30%), peaches (36.8%), tangerines (44.4%); there were reduced amounts of iron in bananas (55.7%), grapefruit (85%), oranges (75%), peaches (78%), strawberries (62%), tangerines (75%), and watermelons (66%); and bananas (57.4%), grapefruit (87.5%), peaches (59.8%), pineapples (55%), strawberries (67.1%), apples (41.1%), and watermelon (38%) lost vitamin A. Ficco et al. [26] observed that the Mg content of fruits decreased by 7 to 25 percent and vegetables by 15 to 35 percent. Bruggraber et al. [27] observed a significant decrease of 0.35 mg 100 g^−1^ (−95%) of iron from the 1930s to 1980s in fruits. Apparent declines in copper from −34 to −81 percent signify minute absolute changes as 100 g^−1^ dry produce of vegetables has a gigantic natural range from 0.11 to 1.71 mg (1555%); in fruit, it varies from 0.10 to 2.06 mg (2060%); and in grains, 0.1–1.4 mg (1400% range) along with its copper availability is hugely subject to the nutrient dilution effect [28]. A sharp decline in the nutrient content of fruits and vegetables was also reported during 1975 to 1997 (Table 1).

## 3. Causes of Declining Nutrient Density

The mineral composition of fruits, vegetables, and food crops is dependent on the genetic make-up of the crop species, climatic circumstances, soil qualities including microbe diversity, management practices, and the extent of ripeness of the plant at harvesting [29,30,31]. The principal causes of the nutrient decline are the degradation of the soil in which crops are grown; developing new high-yield varieties; agronomic factors associated with the commercialization of agriculture; the use of synthetic fertilizers, pesticides, and herbicides to boost food production; improvements in irrigation and the advent of affordable technologies; the introduction of genetically modified food; enhanced air and water pollution; global warming; thinning of the ozone layer; and elevated CO_2_ concentration [28]. The global environmental disaster, including current farming methods with a sole focus on crop yields, has resulted in a decline in crops’ nutrient quality from the baseline values [32]. Modern farming methods are also associated with declines in soil quality, soil microbial diversity, soil water contamination, and the exhaustion of soil nutrients [33,34,35]. There is evidence that the soil quality, the forms and levels of applied nutrients, and the farming system affect the yield and the phytochemical and nutritional composition of produce [36]. An additional consideration is that warming air temperatures and increased solar radiation may lead to higher soil temperatures, resulting in more microbial activity, higher soil respiration rates, and potential limitations in soil nutrient availability. The following important factors are responsible for the declining nutrient density of modern foods.

### 3.1. Alteration in Food Composition

Before the advent of the green revolution and industrialization, conventional foods consisted mainly of naturally grown and cultivated millets such as pearl millet (*Pennisetum glaucum* L.), maize (*Zea mays* L.), and sorghum (*Sorghum bicolor* L.); minor millets such as finger millet (*Eleusine coracana* L.), proso millet (*Panicum miliaceum* L.), foxtail millet (*Setaria italica* L.), kodo millet (*Paspalum scrobiculatum* L.), little millet (*Panicum sumatrense* L.), and barnyard millet (*Echinochloa frumentacea* L.); traditional fruits and vegetables, for example, wild date palm (*Phoenix sylvestris* L.), sitaphal (*Annona squamosa* L.), ber (*Ziziphus* sp.), khirani (*Manilkara hexandra*), jamun (*Syzygium cumini*), pilu (*Salvadora persica*), ker (*Capparis decidua*), lasoda (*Cordia dichotoma*), date palm (*Phoenix dactylifera* L.), tamarind (*Tamarindus indica* L.), cluster bean (*Cyamopsis tetragonoloba* L.), snapmelon (*Cucumis melo momordica*), kachri (*Cucumis calosus*), and leafy vegetables; root staples such as sweet potato (*Ipomoea batatas*); and grains such as beans and barley (*Hordeum vulgare*), which are full of minerals, vitamins, and health-promoting substances such as phenols and antioxidants. With time, some foods became popular, and several are disliked on the basis of the taste, flavor, texture, and appearance of foods; nowadays, the mass population has shifted towards the consumption of less nutritious fast food and packed and imported processed foods. There is increased consumption of unhealthy processed foods accompanied by a neglect of traditional diets [37]. Several underutilized nutrient-rich food crops that were an integral part of family food baskets in the past are gradually being replaced by less nutritious advanced cereals, such as wheat, rice, and maize [38]. Traditional fruits and vegetables are quite superior to modern ones in terms of protein, minerals (Fe, Zn, Ca, Mg, P, and K), fiber, and vitamin B (niacin, vitamin B_6_, and folic acid), and they are also rich in health-promoting phytochemicals, namely, polyphenols, lignans, phytosterols, phytoestrogens, and phycocyanins [14,39]. Similarly, pearl millet, sorghum, and minor millets have a higher content of micronutrients such as calcium, iron, zinc, riboflavin, and folic acid than rice, wheat, and maize [38], which is almost kicked out from the modern food system. In Rajasthan (India), before 1960, the average daily diet consisted of 13 percent minor millets, 13.2 percent sorghum, 19.3 percent pearl millet, 36.5 percent maize, 4.5 percent barley, 1 percent wheat, 5.5 percent pulses, 3 percent meat, 1.5 percent dairy products, 0.5 percent sugar and oil, and 5.5 percent underutilized fruits and vegetables, and there was no consumption of rice or modern fruits, whereas wheat was consumed rarely on the occasion of specific festivals and on the arrival of guests [40]. After the 1980s, there was a drastic increase in wheat, rice, and potato consumption and slight increases in meat, dairy products, sugar, oil, and modern fruit. Similarly, during the 1970s in India, instead of meat and meat products, whole-grain cereals, pulses, fruits, and vegetables were more often consumed [41]. The authors also found significant changes in food composition and a radical increase in wheat consumption (5500%) of tribals in Rajasthan during the last 60 years (Table 2). The most undesirable feature of this nutritional transition is the substitution of millets with socially more prestigious and more refined grains and reduced diversified diets that meet the livelihood but not the nutritional requirement of an individual and are primarily associated with micronutrient deficiency [42]. Hence, it is well reported that alterations in the consumption of various millets, fruits, and vegetables are directly associated with the nutritional health of the habitat.

### 3.2. Growing High-Yielding Varieties

Over the past 60–70 years, plant breeders and physiologists emphasized increasing crop yields through advanced plant genetics tools and intensifying agricultural production systems, and they highlighted the attention not given to maintaining nutritional quality, especially the micronutrient content in crops, which is also essential for the healthy life of the habitat. Modern varieties of fruits, vegetables, and food crops are less nutritious than historically lower potential varieties grown before 1960. A negative correlation between the yield and quality attributes of foods was observed by many researchers, and the high yield in a raised N-system significantly decreased in its concentration of mineral nutrients, nutraceutical compounds, sensory attributes, and the distinctive taste of foods due to the dilution effect [28,43], and considerably increased in carbohydrate [7,20]. Breeding for the traditional metric of yield might result in a reduction in nutritional value [44,45] and also less desirable organoleptic properties [46]. Today, most of the varieties are bred to improve their productivity and profitability and that focus on yield is largely ignoring the nutritional quality of the crops [47]. Too much readily available N, less accessibility to micronutrients, regular irrigation facilities, and intensive agricultural practices have depleted micronutrients from the soil, which obviously tends to diminish the nutrient density of crops [48,49]. A significant difference in mineral content between cultivars has been observed in many horticultural crops including potato, tomato, cucurbits, raspberry, and broccoli [21,44,50]. Traditionally grown tomatoes, cucurbits, okra, and chili were extremely low-yielding, but contained higher nutrient density, distinct taste quality, and organoleptic properties [14]. Marles [28] argues persuasively that any small impact of nutrient dilution from irrigation and fertilizer use is offset by the increased yield that occurs, allowing more people to benefit from the production system, only for the fulfilment of food requirement and not nutrition. So, it is very important to maintain the nutritional quality of foods with respect to increasing yield to mitigate hidden hunger without increasing the availability of foods.

### 3.3. Climate Change and Elevated Carbon Dioxide (eCO_2_)

**Figure 1:**
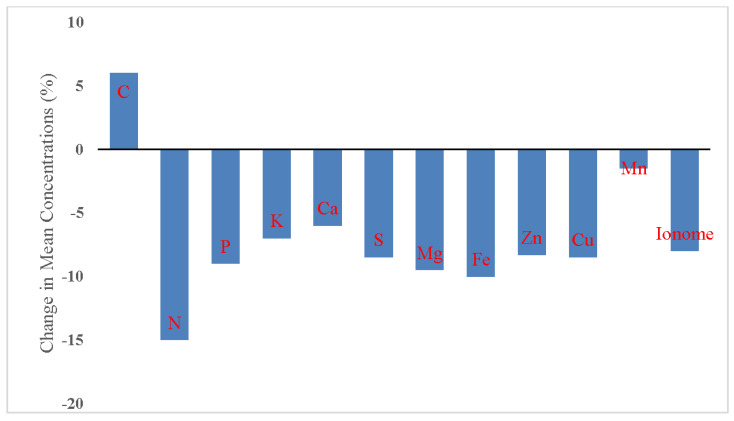
Elevated CO_2_ (eCO_2_ = 689) reduced the overall mineral content of C_3_ plants.

Environmental CO_2_ concentration is increasing globally: the average value of atmospheric CO_2_ concentration was 317 ppm in 1960 and surpassed 400 ppm in 2015, likewise aggressively accelerating the trajectory towards reaching a CO_2_ concentration of 550 ppm by roughly 2050 [51,52]. Climate change and elevated atmospheric carbon dioxide (eCO_2_) affect the availability and quantity of nutrients such as nitrogen, phosphorus, potassium, and iron in soil [53]; decrease nutrient uptake by crops; and reduces proteins in food crops [42,53,54,55,56,57] and mineral nutrient concentration in fruits and vegetables [49,56,58]. Since 1850–1900, carbon dioxide concentrations have risen about 50 percent, which increase photosynthesis and plant biomass production, but also reduce the nutritional quality of crops [49,58,59,60]. Most commonly, decreases in the concentration of xanthophylls, carotenoids [56,61], folate, iron, zinc, vitamins, protein, minerals [1,42,49,59,62,63,64,65], sulfur, methionine, cysteine [66], and the essential sulfur-containing amino acid methionine [67], as well as around 30 percent of the world population suffering from zinc deficiency, are due to elevated atmospheric CO_2_ concentration [68]. However, they are also caused by the immobilization of nitrogen in vegetative tissues and soil [69] and the direct reduction in nitrate assimilation by elevated CO_2_ [70]. The raised CO_2_ concentration in the environment reduced the overall quantity of twenty-five minerals in plants, including calcium, potassium, zinc, sulfur, copper, and iron (Figure 1), by 8 percent on average, and also increased the ratio of carbohydrates to minerals in food plants [49]. Dong et al. [63] observed that eCO_2_ decreased the concentrations of nitrate, protein, and magnesium by up to 9.2 percent, zinc by 18.1 percent, and iron in leafy vegetables (31%), fruit vegetables (19.2%) and root vegetables (8.2%), and also decreased the total antioxidant capacity in fruits and vegetables [71].

### 3.4. Excessive Use of Agrochemicals

During the pre-green revolution era (before the 1940s), food crops were grown organically, but after the 1980s, the maximum number of farmers broadly used insecticide, fungicide, herbicide, and chemical fertilizers. The imbalance in the use of agrochemicals poses a negative effect on the nutritional and organoleptic quality of fruits, vegetables, and food crops. The long-term excessive use of fertilizers may reduce soil organic matter and pollute the soil and underground water with nitrate, and it is consequently hazardous to humans or livestock health. Excessive use of agrochemicals harmfully affects on soil microbial activities and biochemical reactions. The modification of the diversity and composition of the beneficial microbial community can be adverse to plant growth and development either by tumbling nutrient availability or by raising disease incidence [72]. Pesticide contact occurring by means of dermal, digestive, or respiratory routes results in decreased lung function [73], wheezing, higher incidences of lung cancer [74], chronic bronchitis and chronic obstructive pulmonary disease [75], asthma, coughing, rhinitis, and other respiratory symptoms [76]. Bhandari [77] reported that agrochemicals are a reason for serious health hazards and may encourage cancer and certain pesticides to affect the human immune and endocrine systems. Organophosphate insecticides used in vegetables are steadily deposited into the human body and have a link with cancer [78]. In terms of human health, several pesticides cause many kinds of cancer, lung damage, neuronal disorders, birth defects, acute and persistent injuries to the nervous system and reproductive organs, and degenerative diseases; some affect fetal growth and cause congenital anomalies and dysfunction of the immune and endocrine systems [79]. The overuse of synthetic fertilizers and animal waste in crop-growing fields causes the runoff of excess nutrients, which can leach into waterways, exacerbating algae growth in water systems; these algae produce the neurotoxin domoic acid, and eating mollusks contaminated with these strains can cause death [80]. Crop field environments contain a variety of inflammatory aerosols that may increase the risk of lung inflammation and disease in exposed individuals [81]. All these chemicals and environmental pollutants may cause different physical problems in the human body, which also reduce the activities of the digestive system and the absorption of nutrients from foods.

### 3.5. Changes in Agricultural Practices

There is increased attention worldwide towards regaining traditional agricultural practices as a climate-smart approach [82] for helping to produce nutrient-loaded crops. Crops grown in open fields allow different biotic and abiotic stress which accumulate different secondary metabolites and health-promoting compounds, whereas crops grown in protected and plant-congenial conditions have increased yield and contain more water but have reduced nutritional quality due to the dilution effect [83]. The phytochemicals and nutritional quality of fruits, vegetables, and food crops can both increase and decrease as a function of biotic and abiotic stress around the plant grown [84] and abiotic stresses such as high-saline soils, severe drought, and excessive temperatures can harshly change the mineral composition of food crops [85]; similarly, naturally grown, underutilized medicinal crops (*Aloe vera*, *khimp*, and cucurbits) in the western *Thar* desert of Rajasthan have numerous phenols, alkaloid, vitamins, minerals, and protein-rich nutraceutical compounds in surprising quantity as compared to artificially cultivated crops [14]. The key causes for nutrition dilution in vegetable crops is the revolutionization in the varieties and changes in agricultural practice such as protected cultivation [28,83], the usage of higher doses of fertilizers, and irrigation. An extreme downturn in soil physical and biological quality due to certain modern agricultural practices may result in lower nutrient density in fruits, vegetables, and food crops [86,87]. Traditionally, these crops are grown in fields with balanced nutrition, whereas, at the present time, tomatoes, peppers, and cucumbers are produced in soil-less culture such as hydroponics under protected conditions which are optimized to maximize the yield, using artificial fertilizers and irrigation. The nutritional value of the produce is not considered, whereas crops grown in soil may be able to take up some micronutrients in greater amounts through biochemical processes and rhizospheric microorganisms. Plants grown in natural soil may be able to take up many other micronutrients that are not essential for plants but are highly useful for human nutrition [88]. Moreover, there is a surprising lack of robust research that compares the nutritional composition of the crops grown in natural soil and hydroponics (soil-less culture), which increases production incessantly. Farias et al. [89] reported that potatoes cultivated in a vineyard field where copper fungicides had been usually used for a long time had a high intensity of copper in the tubers. Photosynthetic rates may also be important, as plants quickly release photoassimilated carbon to the field soil by means of direct root exudation and associated mycorrhizal fungi, resulting in improved nutrient availability for the plant [90]. It is summarized on the basis of available research that the food crops grown using traditional methods in natural organic soil are rich in microbial diversity and always produce more nutritious and better-quality products.

### 3.6. Postharvest Handling and Storage

Traditionally, most fruits and vegetables were eaten fresh or after minimal processing, which accommodated the original nutritional quality [83]. There have been considerable changes in chemical composition, color, texture, and flavor, and the nutrient dilapidation of fresh fruits and vegetables may also occur during the supply chain and postharvest handling [83,91]. The alteration in nutrient composition from harvest to utilization depends to a certain degree on the specific nutrient, the nature of the commodity, the methods of postharvest management, the storage conditions, and the cooking states [83]. Nutrient retention is optimized if fresh fruits and vegetables are gently handled and stored at high relative humidity with a low temperature [83]. In general, water-soluble nutrients such as vitamin B, vitamin C, and polyphenols are degraded by processing treatments and may be leached into cooking water or the canning medium [83]. Vitamin A, vitamin E, carotenoids, and lycopene are highly responsive to temperature, oxygen, pH, and light and may be released from their cellular matrices by freezing, thermal or high pressure, or additional preservation treatments [83]. Vitamin C is water-soluble and extremely sensitive to high temperature, light, and oxygen, making it susceptible to loss during the cooking of fresh fruits and vegetables and the thermal processing range from 15 to 55 percent; it decreases by 10–90 percent during the canning process. Losses of vitamin C range from 15 percent in green peas to 77 percent in green beans stored at 4 °C for 7 days, whereas losses following 7 days of storage were insignificant at 0 °C but 56 percent at 20 °C in broccoli [91]. The B vitamins are highly responsive to heat and light, and experimental losses as a result of canning range from 7 to 70 percent, and during freezing 20 to 60 percent, from various vegetables. Polyphenolics normally diminish with the storage of fresh peaches, pears, apples, and vegetables as a result of the removal of the skin, canning, and blanching [92]. There was a 10 percent increase in beta-carotene content in carrots and a 10 percent loss in green beans as a result of refrigerated storage for 14–16 days [92]. Lycopene content increased in processed tomato products, probably due to the heat-induced release from its cellular matrix [93]. Processing destroys the naturally occurring enzyme myrosinase that produces nutritious isothiocyanate compounds in Brassica crops. Different vitamin and mineral concentrations in raw fruits and vegetables are slightly affected by the processing methods [91] although trimming may remove plant tissues that are rich in minerals [94]; the removal of soil particles affected the mineral content of spinach and carrots, resulting in small losses of iron.

### 3.7. Decline in Nutrient Concentration in Arable Land

**Figure 2:**
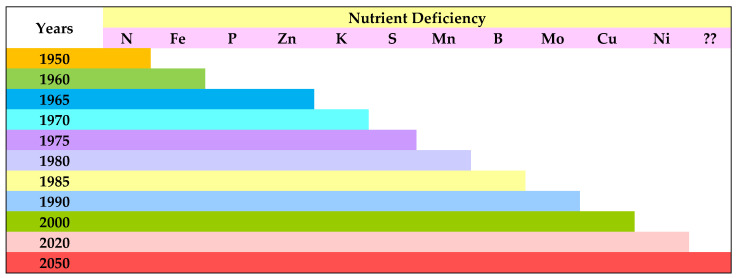
Appearance of nitrogen and micronutrient deficiencies in cultivable land in India.

As we know, arable land all over the world is decreasing day by day due to the adverse effects on nutrient density and microbial diversity and the disruption of soil biological processes and soil physical quality due to the existing agricultural system based on the overuse of agrochemicals [28,95,96] and poor-quality irrigation water [83]. High-potential varieties of rice and wheat increased food production during the green revolution era, which led to a loss of distinctive indigenous crops from nurturing and also caused extinction. The soil degradation activities such as erosion and loss of soil organic matter, soil structure, and soil life influence soil health and quality [97]. Hence, a severe nutrient discrepancy in nitrogen, phosphorus, and potassium occurred widely in the rice and wheat production systems in Asia, Central and South America, and Africa, and globally, the shortage of nitrogen, phosphorus, and potassium was 175 Mha (57% of the cultivated area) for N, 266 Mha (86%) for P, and 283 Mha (91%) for K in developing countries; 31 Mha (69%) for N, 32 Mha (70%) for P, and 31 Mha (69%) for K in the least developed countries; and 108 Mha (52%) for N and 151 Mha (73%) for P in developed countries [98]. Insufficient supply, as well as the unavailability of essential nutrients, is the principal cause behind nutrient stress in our food crops [99]. Singh [100] observed that 49 percent of arable land in India is potentially deficient in zinc, 12 percent in iron, 5 percent in manganese, 3 percent in copper, 33 percent in boron, and 11 percent in molybdenum. Currently, the predictable view of crop nourishment is being reframed around biologically intervening plant–soil interactions [101]. Since then, advances in soil ecology enabled the discovery that soil health plays a major role in building and sustaining soil fertility [102] and the nutritional qualities of food. Over the past century, the topsoil was entirely eroded from about a third of the US Corn Belt [103] and postcolonial farming practices reduced soil organic matter by approximately half [104]; cultivable land degradation already negatively affects the well-being of more than three billion people globally [105]. Alarming conditions were reported by Singh [100] about nitrogen and micronutrient deficiencies in Indian soil due to the mismanagement of soil fertility and health (Figure 2).

### 3.8. Lack of Knowledge about Nutrient-Rich Crops

Since ancient times, traditional foods such as underutilized fruits, vegetables, and millets have been grown organically; they have been important sources of nutrients, dietary fiber, protein, phytochemicals, and vitamins [14] and played a significant role in human nourishment. The traditional wisdom for the cultivation and consumption of these conventional foods decreases significantly with time because of the transformations in food habits and crop cultivation methods [14,16,106]. Indigenous knowledge is a fundamental source for the conservation of the majority of traditional food crops and farming ecosystems [107]. Due to the loss of this aboriginal awareness, the existing generation of farmers and rural youths is not conscious of how to cultivate these ancient food crops and of their role in agrodiversity conservation [108].

## 4. Management Strategy for Maintaining the Nutritional Density of Foods

**Figure 3:**
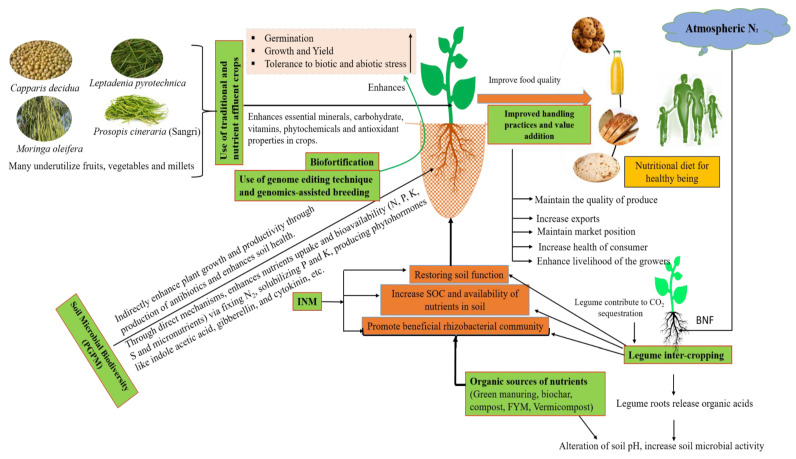
Conceptual overview illustrating the role of various aspects, such as the use biofortified crops, soil microbial biodiversity (PGPM), organic farming, integrated nutrient management, reviving traditional foods, using improved handling practices and value addition, the use of the genome-editing technique and genomics-assisted breeding, restoring soil function, etc., can enhance the nutritional density of crops. Through the various approaches, i.e., transgenic, conventional breeding, and agronomical approaches, the use of the genome-editing technique and genomics-assisted breeding enhances the nutritional quality of food crops. Soil microbial biodiversity increases the bioavailability of nutrients through direct and indirect mechanisms, which improve plant vitality and resilience to biotic and abiotic stress and, ultimately, lead to better nutrient uptake; legumes and INM alter the pH of the rhizospheric soil, which hastens the activity of microorganisms and, thus, regulates the nutrient-cycling process. Increased SOC; total N, P, and K; and organic sources of nutrients (green manuring, biochar, compost, FYM, and vermicompost) increase soil microbial activity and enhance the bioavailability of nutrients and soil health. The use of traditional and nutrient-rich crops enhances essential minerals, carbohydrate, vitamins, phytochemicals, and antioxidant properties in crops; improved handling practices and value addition maintain the quality of produce and increase the health of consumers.

There has been a historical alteration in the nutritional values of conventional fruits, vegetables, and foods, particularly highlighted by dilemmas in countries with a high prevalence of micronutrient malnutrition. There are a number of recommended ways to reduce malnutrition, such as revitalizing traditional food crops, soil nutrient management, adopting organic farming, improving soil microbial diversity, and the biofortification of food crops. Community nutrition programs are focused on an especially inadequate range of micronutrients such as iron, iodine, and zinc; they should give attention to the comprehensive diversity of nutrients that could be lacking through food-based approaches. Plant breeding should aspire to obtain a better stockpile of essential nutrients and minerals, and not just be targeted to solitary nutrients at a time, as is the existing approach of biofortification programs. Farmers, agriculture scientists, and others in the food system are required to be supplied with practical information to recover the nutritional excellence of fruits, vegetables, and food crops. Even so, additional research is required to fill the gaps in knowledge concerning how to recover the nutritional quality of modern foods. The occurrence of the genetic dilution effect should be investigated alongside field trials comparing the nutritional value of modern and traditional varieties. Similarly, to observe the outcome of agronomic dilution, side-by-side trials of the same variety could be undertaken whilst varying the agronomic conditions to increase yield. Long-term experiments of organic versus conventionally grown food crops are still required, using approved protocols. Dietary diversification, supplementations of micronutrients [11,12], improving the nutritional quality of food, biofortification, soil fertility management including plant-breeding approaches [13], promoting natural farming, reviving traditional foods [14], the production of nutrient-intense underutilized fruits and vegetables [15], the promotion of beneficial soil microbial inoculation, and maintaining soil biodiversity [16,17] are the possible ways to combat the nutrient dilution effects of fruits, vegetables, and food crops under the changing climate. Nutrient-rich foods are solutions to fulfill nutrient requirements without overconsuming dietary energy. In this way, the integration of all the above-mentioned technologies in a balanced way is compulsory to minimize the international malnutrition challenges for future generations as well as play an important role in combating the consequences of climate change (Figure 3).

### 4.1. Reviving Traditional foods for Sustainable Nutritional Security

The green revolution transformed production patterns all over the world through promoting high-yielding varieties of rice and wheat and, right now, both crops contribute three-quarters of India’s total cereal production and consumption. These less nutritive cereals increased dramatically with the cost of nutrient-rich millets and sorghum [14,109], which is the principal cause of undernourishment. Indian traditional foods are nutrient-rich, diversified, and also accepted as functional foods because of the presence of therapeutic chemicals, antioxidants, dietary fibers, and a series of minerals and probiotics, which can be helpful for mass body control and blood glucose level and maintain the immune system. In spite of their significance, traditional foods have been vanishing as a result of globalized phenomena, post-green-revolution effects, and less economic compatibility. Today, our food security depends on fewer than ten crops [110] and there has been a steady reduction in the dietary intake of all food groups including millets, legumes, traditional fruits, and vegetables since 1975 [40]. This homogenization of crop cultivation and the related abridged diversity in diets contributed to lower per-capita supplies of key nutrients such as iron, zinc, and vitamin A [109,111] from habitual cornerstones of the native diet, providing essential proteins, vitamins, minerals, and amino acids [14]. Sustainable food security depends on broadening the range of cultivated crops and must include the traditional crops such as millets and underutilized fruits and vegetables [14,112]. Millets have excellent nutritional value compared to modern cereals [113]; good protein, carbohydrates, and dietary fiber; and better amino acid profiles, vitamin A, minerals, starch composition, and phytochemicals including vitamin E, magnesium, and folate, with a low glycemic index [110,114]. In the past, finger millet was a major food component of Rajasthan. It has abundant protein, iron, phosphorus, fiber, and vitamins; is an extraordinary source of calcium (344mg 100g^−1^), which is higher than all cereals; and its iodine content is the highest among all the food grains [115,116]. Various minor and major millets are rich in essential minerals, carbohydrate, vitamins, phytochemicals, and antioxidant properties in comparison to modern cereals (rice and wheat) (Table 3) [117]. Traditional fruits contribute significantly to maintaining nutrition, especially as a very good source of all vital nutrients (Table 4) [117], and contain 27 percent more vitamin C [116], protein (96.55%), fat (98.99%), carbohydrate (75.29%), ascorbic acid (62.94%), thiamin (99.90%), riboflavin (99.94%), niacin (99.21%), minerals (98.63%), calcium (27.07%), phosphorus (55.0%), iron (96.84%), and fiber (97.42%) than modern fruits [117]. Traditional vegetables are also a very good source of different nutrients such as protein (73.21%), fat (74.17%), carbohydrate (18.85%), ascorbic acid (52.24%), energy (18.85%), carotenes (41.59%), thiamin (19.19%), riboflavin (71.33%), calcium (90.12%), phosphorus (47.01%), iron (82.01%), and fiber (89.24%), which are higher in comparison to modern vegetables (Table 5). The important habitual ancient food crops (millets) have higher protein content than rice and wheat, as well as dietary fibers, iron, zinc, calcium, phosphorus, potassium, vitamin B, and important amino acids. Pearl millet is a good source of carbohydrate, energy, dry matter (92.5%), fat (5–7%), ash (2.1%), dietary fiber (1.2g/100g), crude protein (13.6%), quality protein (8–19%), starch (63.2%), a-amylase activity, minerals (2.3mg 100 g^−1^), vitamin A and B, antioxidants, and essential amino acids. It is rich in unsaturated fatty acids (75%) that are useful in lowering cholesterol and reducing cancer risk. Being gluten-free, it is extremely useful for people suffering from celiac diseases. Pearl millet is exceptionally useful for people suffering from diseases such as diabetes, obesity, heart problems, atherosclerosis, and metabolic diseases due to its health-beneficial properties. Due to its excellent nutritional properties, pearl millet is designated as a nutricereal by the Govt. of India (Gazette of India, No. 133 dated on 13 April 2018) for production, consumption, and trade. Similarly, Nandal and Bhardwaj [39] reported that traditional fruits, vegetables, and food crops such as *ber*, *bael*, *ker*, *phog*, *khimp*, *khejri* pod, cluster bean, bitter gourd, *kachri*, and millets are more nutritious than modern food crops and commercial fruits and vegetables. Their critical nutritional value has made these foods highly significant in communal and societal norms and allowed them to stand the test of time [118]. Traditional foods, as well as underutilized fruits and vegetables, often play a significant role in the local economy and nutrition, providing food security, economic stability, and high nutritional quality to those that grow and/or consume [14]. The traditional perception about the processing of foods, their preservation techniques, and their medicinal effects has been established for several generations in India [119]. Therefore, it is the right time to rediscover and reimplement traditional foods to improve the socioecological integrity of agroecosystems and nutritional security. An enhanced right to the use of nutrient-rich millets, fruits, and vegetables was facilitated by their inclusion in the nation’s public distribution system (PDS) that provides subsidized grains to nearly two-thirds of the country’s population. Underutilized food crops should be given equal significance to modern cereals, such as high-productive rice, wheat, and hybrid maize, in national policies and programs [120]. A sufficient supply of good-quality seeds, germplasm, and guidelines on traditional production techniques can be provided to farmers through agricultural extension services with some incentives to farmers in the form of subsidized inputs and mechanisms to support the price, which will generate interest among farmers in cultivating nutritious underutilized food crops. Ultimately, supporting local food chains and processing will recover the awareness of farmers towards cultivating and marketing these indigenous crops, which have the potential to be profitable cash crops [121]. Furthermore, nutrition policies and programs have not focused on creating awareness among communities about the significance of dietary diversity and the inclusion of time-honored food crops in the daily diet as compared to less nutritious fast foods [122]. Nutrient content of traditional food and comparison of the nutritive value of modern and traditional fruits are presented (Table 3 and Table 4) [123]. Most importantly, nutrition education programs aiming to encourage natural farming and traditional diets should be considered in light of the increasing scientific evidence on the therapeutic and medicinal properties of traditional foods. Indigenous knowledge on traditional recipes and preservation methods have eroded in the present era due to modern sociocultural systems. Now, looking at the importance of the nutrient-enriched traditional food crops, renewed focus on the promotion of these crops is needed, which are being relabeled as future-smart foods in view of their high potential for nutrition security, market value, climate resilience, and agrobiodiversity. In this regard, the Government of India has declared the year 2018 as the “Year of Millets” and the United Nations General Assembly (UNGA) has declared the year 2023 as the “International Year of Millets” to bring millets into the mainstream for exploiting their nutritional properties and promoting their cultivation and use.

### 4.2. Integrated Approaches for Soil Nutrient Management

Crop-growing soil and media are basic components for managing the nutrient density of food crops with diversified components. Successful crop production requires only sixteen essential nutrients, but plants grown via natural farming absorb more essential and nonessential mineral nutrients and many other bioactive compounds which are highly useful for increasing the nutraceutical values of fruits, vegetables, and food crops. These bioactive compounds and minerals synthesize different phenolics, antioxidants, and other phytochemicals, which are responsible for increasing the nutritional value of the produce. The integration of different soil management approaches is an emerging need for nutrient-dense food production, such as: soil fertilization and recovering soil biodiversity [124,125], zero-tillage farming or low-till farming [126], microbial inoculation [86,127], increasing earthworm density, the use of biodiverse cover crops, adopting crop rotation, green manuring, or livestock incorporation [128], and land management practices [129,130,131]. The addition of soil amendments for correct soil reactions, recycling farm waste, and returning it to the soil can help the fixation, solubilization, and supply of the nutrient from the soil to plants. These practices have been shown to enhance soil organic matter, obtain better water-holding capacity, and have a significant impact on the amount of carbon sequestration, which could help to mitigate the effects of nutrient density in different food crops. Legume-based intercropping plays many crucial roles such as reducing the requirements of agrochemicals, stimulating biodiversity, altering the pH of the rhizospheric soil, carbon sequestration, restoring soil health [132,133,134], contributing to improving nitrogen and phosphorus fertilizer use efficiency, and leading to the increased availability of phosphorus, iron, and zinc in plants [132,135], which hastens the activity of microorganisms and, thus, regulates the nutrient-cycling process and increases the soil organic carbon and total nitrogen, phosphorus, and potassium. There is an urgent need to assess the hot spots of multinutrient deficiencies, make a contingency plan with diversified components, and create awareness amongst farmers for diagnosing nutrient disorders precisely in order to meet the future demand for nutrients in soils, plants, and humans.

### 4.3. Adopting Organic Farming

The demand for organically grown fruits, vegetables, and food crops is increasing incessantly [136] day by day, owing to their nutritional and health benefits [14]. Presently, consumers are estranged by nutritional scandals from products manufactured using industrial methods and seek safe and prescribed food products [137]. The organic cultivation of food crops is a holistic approach, which is caring to the environment [138]; boosts the natural, chemical, and physical properties of the soil in order to optimize the nutritional quality of food crops [139]; and has a critical socioeconomic impact on a nation [140]. Organically grown food crops retain higher levels of wellness-promoting nutrients and phytochemicals, those shown to exhibit health-protective antioxidant and anti-inflammatory properties [141]. Several researchers have reported that organically nurtured fruits and vegetables contain more protein, vitamin C, phosphorus, potassium, and calcium, as well as higher-quality secondary metabolites, polyphenols, and other nutraceutical compounds [142,143]; iron, magnesium, and dry matter [116,144,145]; phenolic compounds [146]; and polyphenols, carotenoids, and antioxidants [36,144,147,148,149,150,151,152]. Spinach has 77 percent more iron [153]; spinach, Swiss chard, lettuce, and corn has a lower nitrate content and higher vitamin C [154]; and corn, potatoes, apples, and pears retain 60–125 percent more vitamin C, iron, zinc, calcium, phosphorus, magnesium, and potassium and significantly fewer nitrates relative to conventionally grown crops [155]. Organically cultivated fruit wine contains a higher level of resveratrol [156], and tomatoes contain levels of quercetin and kaempferol which are elevated by 79 percent and 97 percent, respectively [36]. Naturally grown oranges, apple, potato, tomato, and papaya have additional quantities of vitamin C, phenolic compounds, total sugars, and flavonoids compared to those cultivated with the use of agrochemicals [157,158,159,160]. Organically produced tomatoes are further enriched with human-health-promoting nutrients, antioxidant activity, phytochemicals [161], and salicylic acid [162], whereas jambu (Acmella oleracea) fruit had extra total phenolics and carotenoids, and jujube fruit has significantly higher pigments, organic acids, chlorophyll, carotenoid, glucose, and total volatile compounds [163] than in recently cultivated fruits [164]. Baraski et al. [165] carried out a meta-analysis of 343 peer-reviewed publications and observed that the concentration of phenolic acids (19%), stilbenes (28%), flavanones (69%), flavones (26%), anthocyanins (51%), and flavonols (50%) was higher in organic crops. The micronutrient content of organic foods ranged up to almost 50 percent higher, and vegetable and legume content up to 10 percent higher, in beta-carotene, vitamin C, boron, copper, and zinc than conventional produce [166]. Bhardwaj et al. [14] also reported that fruits and vegetables grown in organic fields are more nutritious with good organoleptic qualities. Natural farming methods would be the best solution to overcome nutrient deficiency by improving the bioavailability of macro- and micronutrients on cultivable land [36], adding to the soil organic matter, organic carbon, soil organic nitrogen, macro- and microelements, and biological components [32]. Worthington [155] reported that organically grown vegetables contained significantly higher minerals and vitamin C than presently grown vegetables (Table 6).

### 4.4. Improving Soil Ecosystem and Biodiversity

**Figure 4:**
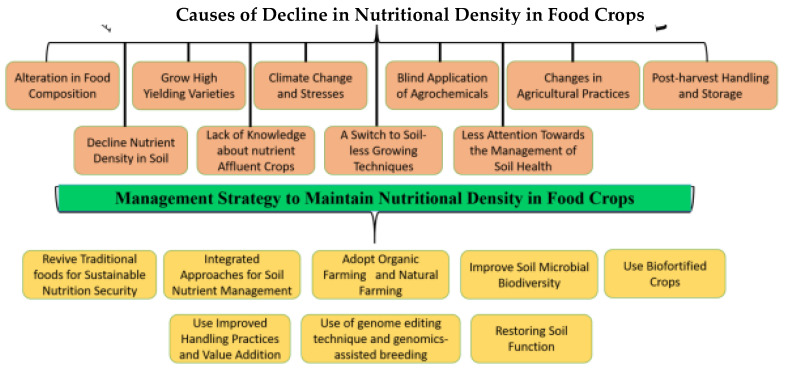
Conceptual diagram showing the main causes for declining nutritional density and strategies for alleviating declining nutritional density of food crops.

Soil biodiversity provides numerous ways of assistance to human health through enhancing crop nutrient uptake and, in this way, obtaining better nutritional significance in foods [167]. Teaspoons of healthy soil have billions of microbes and many individual species. These microorganisms, particularly plant-growth-promoting microorganisms (PGPMs), play a key role in agricultural systems with their diverse mechanisms such as N-fixation, the solubilization of macro- and micronutrients, and the production of phytohormones; antibiotics enhance the availability of nutrients and nutrient uptake [168,169]. Field soil enriched with bacteria and rhizospheric microorganisms has encouraging effects on food’s organoleptic quality and enhances the vitamin, flavonoid, antioxidant, and mineral content [170], whereas specialized soil fungi effectively extend the plant root system with mycelium, releasing nutrients from the soil for plants. However, in modern systems of intensive agriculture involving exhaustive tillage, the use of agrochemicals causes a gradual decline in soil organic matter through the accelerated oxidation and burning of crop residues, causing pollution, greenhouse gas emissions, and the loss of valuable soil–plant system biodiversity, and minimizing the soil microbial population as well as biodiversity. Several studies have demonstrated that PGPR increases the sweetness, moisture content, secondary metabolites, antioxidant properties, content. of minerals, as well as chlorophylls in vegetables and fruits [16,171,172]. Multiple studies have observed that the direct manipulation of the soil microbial population has the potential to enhance the nutritional quality of food crops [173]. The series of studies suggests that orchard soil inoculated with PGPR improves plant nutritional content [174] and crop nutritional and sensory quality [175,176], and increases vitamin C by up to 79 percent in strawberries with the strain Phyllo bacterium sp. PEPV15 [177] and in tomato fruits with the strain Pseudomonas sp. 19Fv1T [178] and plant probiotic Bacillus megaterium and Bacillus amyloliquefaciens [179]. Similarly, the association between arbuscular mycorrhizal fungi (AMF) and plant roots activates the antioxidant, phenylpropanoid, or carotenoid metabolic pathways and enhances the nutraceutical value of horticultural products [180]. Moreover, Gabriele et al. [181] observed that arbuscular mycorrhizal fungi inoculated with fruit wines had better oxidative stability and a considerably higher level of bioactive compounds. Arbuscular mycorrhizal fungi enhance the mineral nutrient uptake of stationary soil nutrients [182,183,184]; increase the concentrations of phosphorus, zinc, copper, and nitrogen in leeks; and increase the zinc, copper, and selenium content in the edible portion of various vegetable crops by approximately 20 percent [185], 35 to 60 percent more antioxidants, vitamins, and significant levels of ascorbic acid and lycopene were found under the organic system with mycorrhizal inoculation in different fruits and vegetables [32]. A series of studies advocate that a decline in the symbiotic relationship with plant roots could, at least partially, clarify the historical declines in the mineral content of fruits and vegetables. Therefore, the use of beneficial microorganisms maintains a better ecosystem with wider biodiversity in field soil for improving the nutritional and nutraceutical quality of food crops (Figure 4).

### 4.5. Using Biofortified Crops

Biofortification is a way of elevating the nutrient density of fruits, vegetables, and food crops from side-to-side conventional and molecular plant breeding, transgenic techniques, and crop cultivation interventions. It is a beneficial and sustainable approach for combating hidden hunger [186] by improving the availability of micronutrients and enhancing vitamin content, essential amino acids, and fatty acid compositions as well as antioxidant levels in food crops [187]. Presently, the thousands of crop varieties available in global seed banks provide biofortified germplasm that can target iron, zinc, copper, calcium, manganese, molybdenum, phosphorus, magnesium, and selenium to produce a higher density of vitamins to generate nutrient-rich breeding lines [188,189]. The alteration rates of β-carotene into vitamin A are high in golden rice and cassava, and maize may integrate an elevated level of nutritional impact [190]. Increasing the quantity of micronutrients in the edible parts of crops, improving bioavailability and absorption in the human body, is also an important way to mitigate nutrient deficiency [191]. More than 20 million people in developing countries are consuming biofortified beans and pearl millet with iron; maize, cassava, and sweet potato with provitamin A; and rice and wheat by means of zinc- [189] and vitamin-A-enriched rice produced using transgenic approaches [192], but all these technologies are limited in commercial fruits and vegetables. Biofortification methods comprise agricultural practices such as increasing micronutrient concentration [188], chemical fertilization, the accumulation of mycorrhizal fungi and nitrogen-fixing bacteria, the intercropping of legumes with grass crops for increased nutrient content in the root zone, and both conventional and transgenic crop-breeding methods [186,193,194,195,196,197]. Minimizing the undesirable effects of climate change on the nutritional quality of food crops can be achieved through biofortified breeding methods in food crops [99]. Most of the underutilized fruit and vegetable crops, uncultivated relatives, landraces, and local cultivars are lower in yield but are a wealthy source of nutrients, which offer their efficient utilization in the crop improvement program for nutrient enrichment [198]. Fortifying rice with iron alone or in combination with other micronutrients has a slight apparent effect on the risk of anemia and vitamin A deficiency [199]. Now, it is an urgent need to develop new varieties of many food crops completely loaded with nutrients through various novel and cost-effective technologies, such as genome editing and genomics-assisted breeding, for the elimination of malnutrition.

### 4.6. Using Improved Handling Practices and Value addition

Postharvest management practices such as harvesting, washing, cleaning, curing, disinfecting, sorting and grading, packaging, storing, and transportation not only play a vital role in maintaining the quality of the fruits and vegetables but also extending the shelf life [83]. Crop-specific postharvest handling practices and treatment protocols can be used for improving the shelf life of food crops. Proper postharvest treatments of fresh produce slow down the physiological processes of senescence and maturation and help in reducing the development of physiological disorders, minimize the risk of microbial contamination, and maintain the nutritional and organoleptic quality of the fresh produce [200]. In developed and fast-developing countries, people prefer ready-to-cook food or ready-to-eat food materials. So, scientists, food-processing industries, and innovators have to develop various recipes and value-added products from nutrient-rich millets and underutilized fruits and vegetables, which will definitely promote the wider acceptance among consumers of different ages and incomes and play a significant role in the nutritional security of future generations.

## 5. Conclusions

Numerous environmental factors, faulty and intensive agricultural practices, as well as a lack of knowledge about nutrient-rich crops can contribute to a decline in the nutritional quality of food crops. Collaborative efforts from the various stakeholders are needed to ensure the integration of different technologies to minimize nutrient deficiency in foods. Improving the nutrient density and consumption of fruits, vegetables, and nutrient-rich foods is imperative to tackling global micronutrient deficiencies, and optimizing the nutritional quality of these foods is important to offer the best chance of meeting the requirements. Now, it is of utmost importance to encourage the use of nutritionally enriched and nutraceutical foods to mitigate the gap and maintain the health of future generations. All this points to the need to reframe nutritional and agricultural research and policy, and further research evaluating the role of soil health on the nutrient density of food is needed to enhance our understanding of the linkages between diet, farming practices, and human health.

## Figures and Tables

**Table 1:** Nutrients’ decline trend (%) in different fruits* and vegetables* during 1975 to 1997.

Fruits	Calcium	Iron	Vitamin A (IU)	Vitamin C	Vegetables	Calcium	Iron	Vitamin A (IU)	Vitamin C
Apples (mg)	None	40.00	41.10	Up 42.50	Broccoli (mg)	53.40	20.00	38.30	17.50
Apricots (mg)	17.70	Up 8.00	3.30	None	Cabbage (mg)	4.10	Up 47.50	Up 2.30	31.90
Banana (mg)	25.00	55.70	57.40	9.00	Carrots (mg)	27.00	28.60	Up 155.70	Up 16.30
Cherries (mg)	31.80	2.50	Up 94.60	30.00	Cauliflower (mg)	12.00	60.00	68.30	40.50
Grapefruits (mg)	25.00	85.00	87.50	12.40	Collard greens (mg)	28.60	81.00	41.20	61.60
Lemons (mg)	57.40	14.30	3.30	31.20	Daikon (mg)	22.90	33.30	100.00	31.30
Orange (mg)	2.40	75.00	Up 2.50	Up 6.40	Kale (mg)	24.60	22.70	None	4.00
Peaches (mg)	44.40	78.00	59.80	5.70	Mustard greens (mg)	43.70	51.30	24.30	27.80
Pineapples (mg)	58.80	26.00	55.00	9.40	Onion (mg)	25.90	56.00	100.00	36.00
Strawberries (mg)	33.30	62.00	67.10	3.90	Parsley (mg)	32.00	None	38.80	22.70
Tangerines (mg)	65.00	75.00	Up 119.0	7.00	Turnip greens (mg)	22.80	38.90	None	56.80
Watermelons (mg)	Up 14.30	66.00	38.00	Up 37.10	Watercress (mg)	20.50	88.20	4.10	45.60
Net Change	28.90	16.40	16.40	1.90	Net Change	26.50	36.10	21.40	29.90

* Based on 100-gram edible portion. Source: USDA food composition tables.

**Table 2:** Alteration in food composition of tribal farmers of Rajasthan during the last 60 years.

Food Groups/Foods	Percent Share in Diet of Tribal Farmers (N = 1500)				
	Before 1960	1960 to 1980	1981–2000	2001 to 2020	%Change
Minor millets *	13.0	6.0	2.5	0.2	−98.46
Sorghum	13.2	9.5	5.8	1.0	−92.42
Pearl millet	19.3	15.0	10.0	5.3	−72.54
Maize	36.5	38.0	20.3	10.2	−72.05
Barley	4.5	6.7	7.2	4.0	−11.11
Wheat	1.0	4.5	30.0	56.0	5500.00
Rice	0.0	1.0	1.8	7.5	650.00
Pulses	5.5	4.5	3.0	2.0	−63.64
Meats	3.0	3.5	4.0	4.8	60.00
Dairy products	1.5	2.5	3.3	3.0	100.00
Sugar/gur and oils	0.5	1.0	1.3	2.0	300.00
Traditional fruits and vegetables	5.5	7.5	6.5	3.0	−45.45
Modern fruits and vegetables	0.0	0.3	2.0	3.5	1066.67
Traditional wine and English wine consumption trend in tribals					
Traditional wine	100.0	99.5	70.5	34.2	−65.80
Modern wine	0.0	0.5	29.5	65.8	6480.00

* Finger millet (*Eleusine coracana* L.), proso millet (*Panicum miliaceum* L.), foxtail millet (*Setaria italica* L.), kodo millet (*Paspalum scrobiculatum* L.), little millet (*Panicum sumatrense* L.), and barnyard millet (*Echinochloa frumentacea* L.).

**Table 3:** Nutrient content of traditional food (millets) and modern foods (cereals) per 100 g.

S.N	Nutrients	Traditional Food (Millets) *												Modern Foods (Cereals) **			% Change
		Pearl Millet	Sorghum	Ragi	Foxtail Millet	Proso Millet	Barnyard Millet	Kodo Millet	Little Millet	Desi Maize	Barley	Oat	Mean	Rice	Wheat	Mean	
1	Protein (g)	11.6	10.4	7.3	12.3	7.7	6.2	8.3	10.13	11.1	11.5	16.9	10.31	7.94	12.1	10.02	−2.83
2	Fat (g)	5.0	1.9	1.3	4.3	4.7	2.2	1.4	4.7	3.6	1.30	6.9	3.39	0.52	1.7	1.11	−67.27
3	CHO (g)	67.5	68.2	72.6	60.9	70.4	65.5	65.9	65.55	64.77	61.29	62.0	65.87	78.24	64.72	71.48	+8.51
4	Energy (Cal.)	361.0	349.0	328.0	331.0	341.0	397.0	309.0	329.0	342.0	336.0	389.0	346.5	345.0	346.0	345.5	−0.30
5	Folic acid (mg)	45.5	20.0	18.3	15.0	9.0	-	23.1	36.20	20.0	31.58	56.0	27.47	9.32	36.6	22.96	−16.41
6	Thiamin (mg)	0.33	0.37	0.42	0.59	0.21	0.33	0.33	0.26	0.42	0.36	0.76	0.40	0.05	0.49	0.27	−32.19
7	Riboflavin (mg)	0.25	0.13	0.19	0.11	0.01	0.10	0.09	0.05	0.10	0.18	0.14	0.12	0.05	0.17	0.11	−10.37
8	Zinc (g)	3.1	1.6	2.3	2.4	3.7	3.0	0.7	1.82	2.8	1.50	4.0	2.45	1.21	2.2	1.70	−30.33
9	Calcium (mg)	42.0	25.0	344.0	31.0	17.0	20.0	27.0	17.0	10.0	26.0	54.0	55.73	10.0	48.0	29	−47.96
10	Iron (mg)	8.0	4.1	4.62	2.8	9.3	5.0	0.5	9.3	2.3	1.67	5.0	4.78	0.7	4.9	2.8	−41.43
11	Phosphorus (mg)	289.0	274.0	268.0	110.0	-	-	-	157.0	279.0	178.0	-	222.1	96.16	315.0	205.6	−7.46
12	Fiber (g)	1.2	1.6	3.6	8.0	7.6	9.8	9.0	7.6	2.7	3.9	11.6	6.05	0.82	1.2	1.01	−83.32
13	Total phenol	67.71	23.25	135.0	106.0	0.10	26.7	368.0	14.24	32.92	23.47	-	79.74	3.14	14.33	8.735	−89.05

* Pearl millet (*Pennisetum glaucum* L.), Sorghum (*Sorghum bicolor* L.), Ragi (*Eleusine coracana* L.), Foxtail millet (*Setaria italica* L.), Proso millet (*Panicum miliaceum* L.), Barnyard millet (*Echinochloa frumentacea* L.), Kodo millet (*Paspalum scrobiculatum* L.), Little millet (*Panicum sumatrense* L.), Maize (*Zea mays* L.), Barley (*Hordeum vulgare*), and Oat (*Avena sativa*).** Rice (*Oryza sativa*) and Wheat (*Triticum aestivum*).

**Table 4:** Comparison of the nutritive value of modern and traditional fruits (100 g edible portion of fruits).

S.N	Nutrients	Traditional Fruits **										Modern Fruits *								% Change
		Karonda	Phalsa	Bael	Khirni	Timru	Sitaphal	Ker	Tamarind	Ber	Mean	Apple	Orange	Guava	Mango	Banana	Papaya	Grape	Mean	
1	Protein (g)	1.15	1.30	1.80	0.50	6.0	1.60	14.24	3.10	1.34	3.45	0.20	0.70	0.90	0.60	1.20	0.60	0.50	0.67	−96.55
2	Fat (g)	1.67	0.90	0.30	2.40	-	0.40	2.00	0.10	0.35	1.02	0.50	0.20	0.30	0.40	0.30	0.10	0.30	0.30	−98.99
3	CHO (g)	2.87	14.7	31.8	27.7	26.8	23.5	18.20	67.4	9.40	24.71	13.4	10.9	11.2	16.9	27.2	7.20	16.5	14.76	−75.29
4	Energy (Cal.)	34.0	72.0	137	134.0	112.0	104.0	107.0	283.0	204.0	131.9	59.0	48.0	51.0	74.0	116.0	32.0	71.0	64.43	+31.89
5	Ascorbic acid (mg)	135.0	22.0	8.0	16.0	1.0	37.0	50.0	3.62	60.93	37.06	3.57	30.0	212.0	16.0	7.00	57.0	1.0	46.65	−62.94
6	Carotenes (μg)	55.89	419.0	55.0	495.0	361.0	-	-	188.0	76.80	235.8	229.0	1104	996.0	2743.0	78.0	666.0	216	861.71	+135.81
7	Thiamin (mg)	0.01	-	0.13	0.07	-	0.07	-	0.34	0.01	0.11	0.93	0.07	0.03	0.08	0.05	0.04	0.04	0.18	−99.90
8	Riboflavin (mg)	0.02	-	0.03	0.08	-	0.17	-	0.07	0.02	0.07	0.01	0.02	0.03	0.09	0.08	0.25	0.03	0.07	−99.94
9	Niacin (mg)	0.25	0.30	1.10	0.70	-	1.30	-	1.56	0.33	0.79	0.25	0.28	0.40	0.90	0.50	0.20	0.12	0.38	−99.21
10	Minerals (g)	-	1.10	1.70	0.80	0.80	0.90	-	2.9.	-	1.37	0.30	0.30	0.70	0.40	0.80	0.50	0.6	0.51	−98.63
11	Calcium (mg)	10.81	129.0	85.0	83.0	60.0	17.0	55.0	170.0	46.55	72.93	10.0	26.0	10.0	14.0	17.0	17.0	20	16.29	−27.07
12	Phosphorus (mg)	32.62	39.0	50.0	17.0	20.0	47.0	57.0	110.0	32.38	45.00	14.0	20.0	28.0	16.0	36.0	13.0	30	22.43	−55.00
13	Iron (mg)	0.87	3.10	0.60	0.90	0.50	4.31	0.76	17.0	0.40	3.16	0.66	0.32	0.27	1.30	0.36	0.50	0.52	0.56	−96.84
14	Fiber (g)	1.38	1.20	2.90	3.0	0.80	3.10	4.24	5.60	1.02	2.58	1.0	0.30	5.20	0.70	0.40	0.80	2.9	1.61	−97.42

* Apple (Malus pumila), Orange (Citrus sinensis), Guava (Psidium guajava), Mango (Mangifera indica), Banana (Musa × paradisiacal), Papaya (Carica papaya), Grape (Vitis vinifera). ** Karonda (*Carissa carandas*), Phalsa (*Grewia asiatica*), Bael (*Aegle marmelos*), Khirni (*Manilkara hexandra*), Timru (*Salvadora persica*), Sitaphal (*Annona squamosa* L.), Ker (*Capparis decidua*), Tamarind (*Tamarindus indica* L.), and Ber (*Ziziphus* sp.).

**Table 5:** Nutrient content of traditional vegetables and modern vegetables per 100 g.

S.N	Nutrients	Traditional Vegetables *										Modern Vegetables **				% Change
		Cluster Bean	Bathua Leaf	Kinkoda	Kachri	Khimp	Khejri Pod	Spinach	Snap Melon	Amaranthus Leaf	Mean	Tomato	Potato	Brinjal	Mean	
1	Protein (g)	3.20	2.50	5.44	0.28	3.13	23.1	2.14	0.37	3.29	4.83	0.76	1.35	1.77	1.29	−73.21
2	Fat (g)	0.40	0.44	3.10	1.28	1.84	0.52	0.64	1.12	0.65	1.11	0.25	0.22	0.39	0.29	−74.17
3	CHO (g)	10.80	2.56	7.70	7.45	9.83	14.15	2.05	15.6	2.28	8.05	3.20	12.9	3.49	6.53	−18.85
4	Energy (Cal.)	16.0	27.71	288.0	43.0	68.0	82.0	24.37	74.0	30.58	72.63	18.87	53.76	27.23	33.29	−54.17
5	Ascorbic acid (mg)	17.92	41.03	-	29.81	39.0	-	30.28	18.6	83.54	37.17	25.27	26.41	1.58	17.75	−52.24
6	Carotenes (μg)	1192.0	3469.0	-	-	-	-	9553.0	-	20.47	3558.62	5826.0	224.0	186.0	2078.67	−41.59
7	Thiamin (mg)	0.05	0.06	0.05	-	-	-	0.16	-	0.01	0.07	0.04	0.05	0.07	0.05	−19.19
8	Riboflavin (mg)	0.03	0.51	0.10	-	-	-	0.10	-	0.19	0.19	0.02	0.01	0.13	0.05	−71.33
9	Niacin (mg)	0.71	0.54	0.06	-	-	-	0.33	-	0.71	0.47	0.51	1.36	0.74	0.87	+85.11
10	Calcium (mg)	130.0	211.0	33.7	0.09	414.0	0.41	82.29	0.76	330.0	133.58	8.90	8.53	22.17	13.20	−90.12
11	Phosphorus (mg)	57.0	37.55	42.0	0.003	317.0	0.05	32.59	0.09	73.22	62.17	15.45	43.42	39.95	32.94	−47.01
12	Iron (mg)	1.08	2.66	5.04	0.18	3.48	0.48	2.95	0.84	4.64	2.37	0.22	0.57	0.49	0.43	−82.01
13	Fiber (g)	3.20	1.68	3.0	1.21	23.18	6.7	0.86	1.34	1.20	4.71	0.30	0.54	0.68	0.51	−89.24

* Cluster bean (Cyamopsis tetragonoloba L.), Bathuwa leaf (*Chenopodium album*), Kinkoda *(Momordica dioica*), Kachri (Cucumis calosus), Khimp (Leptadaenia pyrotechnica), Khejri pod (*Prosopis cineraria*), Spinach (Spinacia oleracea), Snapmelon (Cucumis melo momordica), Amaranthus leaf (*Amaranthus cruentus*). ** Tomato (*Solanum lycopersicum*), Potato (*Solanum tuberosum*), Brinjal (*Solanum melongena* L.).

**Table 6:** The nutrient content in organically grown vegetables in comparison to conventional ones.

Vegetable	Nutrition in Organic Vegetables in Relation to Conventional (%)			
	Vitamin C	Iron	Magnesium	Phosphorus
Lettuce	+17	+17	+29	+14
Spinach	+52	+25	−13	+14
Carrot	−6	+12	+69	+13
Potato	+22	+21	+5	0
Cabbage	+43	+41	+40	+22

## Data Availability

The data is included in the article.
